# The Treatment of Cancer of the Tongue

**Published:** 1881-06

**Authors:** 


### THE TREATMENT OF CANCER OF THE TONGUE.

In the December number of the *Bull. et Mem. de la Soc. de Chir.* (quoted in London *Medical Record*), is a report of a communication by M. Verneuil on the inutility and danger of any save operative treatment of epithelioma of the tongue. This disease, it is pointed out, has never been cured, either by internal treatment or by topical applications. M. Verneuil states that he seldom meets with a case of lingual cancer which has not previously been treated by iodide of potassium. Such treatment, it is argued, cannot be justified. It is a gross error to think that iodide of potassium has any efficacy in instances of true neoplasm, for, as is well known, this agent effects no amelioration in cancers of the lip, cervix uteri, and other organs. The diagnosis of epithelioma of the tongue, in the majority of cases, should not be difficult; and through the recent teaching of M. Fournier this affection may be readily distinguised from tertiary glossitis. There are, it is true, hybrid cases in which the cancer occurs in a subject of old syphilis, and in which the objective characters of the epithelioma are modified by the constitutional affection. This condition does not indicate any alteration of treatment, for the hybrid affection, in its course, termination and prognosis, resembles pure epithelioma, and should be dealt with in a like manner. M.

Verneuil asserts that medicinal treatment of cancer of the tongue is not only fruitless, but also hurtful. Surgical removal of the disease is postponed, and so much precious time is lost. Iodide of potassium often excites troublesome and obstinate glossitis, and mercury stimulates the growth both of the primary epithelioma, and of any affected cervical glands. Chlorate of potash is as useless in cases of cancer of the tongue, as it would be if given for the relief of cancer of the cervix uteri. Epithelioma of the tongue, M. Verneuil holds, if taken in time, may be cured by operation. Menti n is made of four cases of complete cure. In three of these cases the growth was small and superficial? in the fourth case a relapsing growth, together with a degenerated gland, was removed. When more than a third of the tongue has become involved, when the floor of the mouth is also affected, and when the disease has been progressing for longer than a year, the case is deplorably grave, and cannot then be treated by operation with any chance of success. Removal of lingual cancer, it is asserted, is not a serious operation when it can be performed by the mouth, and when the disease is recent and not extensive. Of two hundred such operations performed under these conditions by M. Verneuil, one only was fatal; and, in this instance, the death was due to pneumonia, and not directly to surgical action. Operation at an early stage of the disease is strongly recommended; and it is stated that between the early and favorable stage and the advanced and hopless stage there is a long interval, at any period of which the surgeon may operate without risk, and with some prospect of permanent success.

